# Early Physical Activity and Discharge Destination after Stroke: A Comparison of Acute and Comprehensive Stroke Unit Care

**DOI:** 10.1155/2013/498014

**Published:** 2013-12-04

**Authors:** Tanya West, Leonid Churilov, Julie Bernhardt

**Affiliations:** ^1^School of Health Sciences, La Trobe University, Melbourne, VIC 3086, Australia; ^2^Physiotherapy Department, Royal Perth Hospital, Perth, WA 6000, Australia; ^3^Stroke Division, Florey Institute of Neuroscience and Mental Health, Melbourne, VIC 3084, Australia; ^4^Department of Mathematics and Statistics, University of Melbourne, Melbourne, VIC 3010, Australia

## Abstract

*Background*. Common models of acute stroke care include the acute stroke unit, focusing on acute management, and the comprehensive stroke unit, incorporating acute care and rehabilitation. We hypothesise that the rehabilitation focus in the comprehensive stroke unit promotes early physical activity and discharge directly home. *Methods*. We conducted a two-centre prospective observational study of patients admitted to a comprehensive or acute stroke unit within 14 days poststroke. We recruited 73 patients from each site, matched on age, stroke severity, premorbid function, and walking ability. Patient activity was measured using behavioural mapping. Therapy activity was recorded by therapist report. Time to first mobilisation, discharge destination, and length of stay were extracted from the medical record. *Results*. The comprehensive stroke unit group included more males, fewer partial anterior circulation infarcts, more lacunar infarcts, and more patients ambulant without aids prior to their stroke. Patients in the comprehensive stroke unit spent 14.4% more (95% CI: 8.9%–19.8%; *P* < 0.001) of the day in moderate or high activity, 18.5% less time physically inactive (95% CI: 5.0%–32.0%; *P* = 0.008), and were more likely to be discharged directly home (OR 3.7; 95% CI 1.4–9.5; *P* = 0.007). *Conclusions*. Comprehensive stroke unit care may foster early physical activity, with likely discharge directly home.

## 1. Introduction

Evidence of the benefits of organised stroke unit care for the treatment of acute stroke is now well established [1]. Early physical activity has been identified as a key component of this care [2, 3], with two small randomised controlled trials of very early rehabilitation providing preliminary evidence for the benefits of early physical activity after stroke [4, 5].

The emergence of different models of stroke unit care has created a need for further research which directly compares these different models and examines the underlying components of care [1]. The acute stroke unit (ASU) and the comprehensive stroke unit (CSU) have been identified as common models of acute care for stroke [1]. There are few trials which directly compare these two stroke unit models [6–9] and there is currently insufficient evidence to confirm a greater benefit from either model [1]. In a recent review of the literature describing these two models of care we found that ASU care tends to have a greater emphasis on acute medical management, increased nurse staffing, early assessment and investigation, and intensive physiological monitoring, while CSU care appears to have a greater emphasis on early rehabilitation, multidisciplinary teamwork, and the involvement of patients and carers [10]. These features of CSU care may promote an increased level of early physical activity in comparison to the ASU model of care.

The purpose of this study is to directly compare early physical activity in a CSU and an ASU. The primary aim is to compare the amount and type of physical activity undertaken throughout the day by patients in the first 14 days poststroke. Secondary aims are to compare where and with whom this activity takes place, the amount of formal therapy received, when patients first commence physical activity out of bed, and the length of stay and discharge destination. We hypothesise that patients admitted to CSU care are more active, commence activity sooner, receive more therapy, and will be more likely to be discharged directly home when compared to a similar cohort of patients admitted to ASU care.

## 2. Methods

### 2.1. Study Design, Setting, and Participants

This study was a prospective observational study which took place in the stroke units of two large metropolitan teaching hospitals in Australia. The Austin Hospital is located in Melbourne, Victoria, and its stroke unit is a 13-bed ASU within a neurology ward. The Royal Perth Hospital is located in Perth, WA, and its stroke unit is a 14-bed CSU, also within a neurology ward.

The ASU and the CSU are the most common models of stroke unit care in Australia. In a 2011 audit of Australian stroke services 58 (78%) of the 74 stroke units surveyed were identified as ASUs and 15 (20%) as CSUs [11]. The Stroke Unit Trialists Collaboration defines the ASU as a unit to which patients are admitted acutely and discharged early and which may include intensive monitoring, high nurse to patient ratios, and the potential for life support [1]. Conversely, the CSU is defined as a unit that combines acute care and rehabilitation, admitting patients acutely but also providing a period of rehabilitation if required [1]. Beyond these definitions stroke unit care is often described only in a very general sense in the literature and we have previously identified a lack of information regarding the specific characteristics of each of the different stroke unit models [10]. As such we classified the stroke services based on the clinicians' descriptions' of their units. In accordance with the Stroke Unit Trialists Collaboration definitions of the ASU and CSU models of care [1] both stroke units admitted patients acutely and provided acute care. Patients in the ASU who required inpatient rehabilitation were transferred to a rehabilitation facility at another site. In the CSU rehabilitation was provided simultaneously as part of the acute management and ongoing rehabilitation could be provided for as long as necessary on the stroke unit; however, most patients requiring inpatient rehabilitation beyond a few weeks were usually transferred to a rehabilitation facility at another site.

Eligible patients were aged 18 years or over, with a diagnosis of first or recurrent stroke (infarct or haemorrhage), who were admitted to the stroke unit and were within 14 days of stroke onset. Patients were recruited over a three-year period from January 2008 to December 2010. Patients were excluded from the study if they were receiving palliative care or if discharge was planned prior to completion of the day of behavioural observation.

### 2.2. Behavioural Mapping

Physical activity, location, and people present were recorded across the day for each patient using established standardised behavioural mapping procedures, which have been previously demonstrated to have high interrater reliability [12]. High consistency of patient behaviour across days has been reported in a previous study [13]; therefore, each individual patient was observed for a single working day. Observation days were undertaken approximately every six to eight weeks and up to 10 patients could be recruited for each day of observation. Behavioural mapping was carried out over a nine-hour period between 8 am and 5 pm when the patients were considered to be most active. Observations took place at 10-minute intervals with the exception of up to five randomly scheduled 10-minute rest periods for the observer. Patients and staff were informed that patient activity was being monitored; however, they were instructed that they should not alter their usual behaviour. Wherever possible the observer attempted to remain inconspicuous to avoid influencing behaviour.

Physical activity was grouped into the following five categories based on previous activity definitions [12].

- Nil physical activity: lying in bed inactive.
- Nonphysical activity: passive activities while resting in bed including reading, watching TV, talking, and eating.
- Low physical activity: sitting supported out of bed, hoist transfers.
- Moderate physical activity: sitting unsupported, transfers with feet on floor.
- High physical activity: standing, walking, stair climbing.

### 2.3. Formal Therapy Activity

Treating occupational therapists and physiotherapists provided a self-report of the amount and type of physical activity undertaken by recruited patients during formal therapy sessions on the day of observation. The validity of this method of therapist report has been previously established [14] and may provide more comprehensive information regarding patient activity during therapy sessions than the intermittent behavioural mapping observations alone.

### 2.4. First Mobilisation

The time to first mobilisation, defined as first out of bed activity from both the time of stroke onset and from the time of hospital admission, was derived from the medical record.

### 2.5. Patient Characteristics

Demographic data and information regarding the patient's stroke were acquired from the medical record. Premorbid function was determined using the modified Rankin Scale (mRS) [15]. Type of stroke was classified according to the Oxfordshire Community Stroke Program (OCSP) classification [16]. Stroke severity was determined using the National Institutes of Health Stroke Scale (NIHSS) [17] from a retrospective review of the medical records [18]. The patient's motor function on the day of observation was assessed by the treating physiotherapist using the Mobility Scale for Acute Stroke (MSAS) [19]. The gait score from this scale was used to group patients into independent (MSAS gait = 6) or dependent (MSAS < 6) ambulation categories.

### 2.6. Patient Discharge

Length of stay in the stroke unit and discharge destination from the stroke unit were determined from a retrospective review of the medical record.

### 2.7. Ethics

Approval of this study was obtained from the Human Ethics Committees at the Austin Hospital, the Royal Perth Hospital, and the Faculty of Health Sciences at La Trobe University. Informed consent was obtained from all participants or a responsible third party where the patient was unable to provide consent themselves.

### 2.8. Data Analysis

Unless stated otherwise all statistical analyses were performed using SPSS version 19. To assess differences in patient characteristics between stroke units, numerical data (age, stroke severity, and days poststroke) were analysed using the Mann-Whitney *U* test due to nonnormal distributions, while all other patient characteristics were analysed using Fisher's exact test due to the categorical nature of this data. Initial analyses revealed significant differences between stroke units in multiple patient characteristics; therefore, patients were matched across sites using propensity score matching as implemented in Stata IC version 12, on the basis of age, stroke severity (NIHSS), premorbid function (premorbid mRS > 2), and ambulation status on the day of observation (MSAS Gait < 6). The purpose of the matching was to ensure broad comparability of the patient groups across two sites as far as matching variables are concerned rather than strict individual matching. Therefore, as some potentially significant differences between the groups on other variables could remain, the patients in the two groups were not regarded as fully individually matched, and all the subsequent statistical analyses were conducted on the unpaired basis.

For the behavioural mapping data Microsoft Access 2003 was used to automatically determine the highest category of physical activity recorded for each 10-minute observation period. Medians and interquartile ranges (IQR) are reported for the percentage of time which patients spent in each activity category, in each location, and with different people present. Linear least-squares regression analyses were initially attempted to examine differences between stroke units in the proportion of the day spent inactive or involved in nonphysical activity and in moderate or high level physical activity. However, the data for moderate or high level activity were highly skewed and the assumption of constant variance of the residuals was not met for a linear regression model; therefore, Stata IC version 12 was used to conduct univariate median regression analyses. Multivariable median regression analyses were then performed to adjust for the effect of age, stroke severity, gender, days poststroke, and premorbid function.

The median minutes per day, median minutes per session, and the proportion of patients receiving zero, one, or two sessions per day are reported for physiotherapy and occupational therapy from the therapist report data. The minutes per day of physiotherapy and occupational therapy were compared using the Mann-Whitney *U* test.

The median time to first mobilisation was calculated from the first mobilisation data. Differences between stroke units in the time to first mobilisation were examined using the Mann-Whitney *U* test.

Median length of stay and the proportion of patients discharged to different destinations were determined from the discharge data. Univariate logistic regression analysis was used to examine the difference between units in the proportion of patients discharged directly home. Multivariable logistic regression analysis was undertaken to adjust for the effect of age, stroke severity, gender, and premorbid function.

## 3. Results

### 3.1. Patient Characteristics

Across both units 232 patients were recruited (ASU 93, CSU 139). We excluded 19 patients who were part of a randomised controlled trial investigating very early mobilisation [20], three patients who did not complete the day of observation due to unexpected discharge, four who were more than 14 days poststroke, and two who had already been observed on a previous day. From the remaining 204 patients (ASU 74, CSU 130) we identified 73 matched patients from each site for analysis in the current study. All but one of the unmatched patients were from the CSU and the patient characteristics for the full CSU cohort have been described previously [21]. Patient characteristics for the patients analysed in the current study are summarised in Table 1. Despite the matching process, some statistically significant differences still existed between the participants from each site. In the CSU patient group there were more males (51 CSU; 35 ASU), fewer patients with partial anterior circulation infarcts (PACI's) (19 CSU; 29 ASU) and more with lacunar infarcts (LACI's) (23 CSU; 6 ASU), and more patients who were able to ambulate independently without aids prior to their stroke (64 CSU; 51 ASU).

### 3.2. Behavioural Mapping

#### 3.2.1. Physical Activity

**Figure 1:**
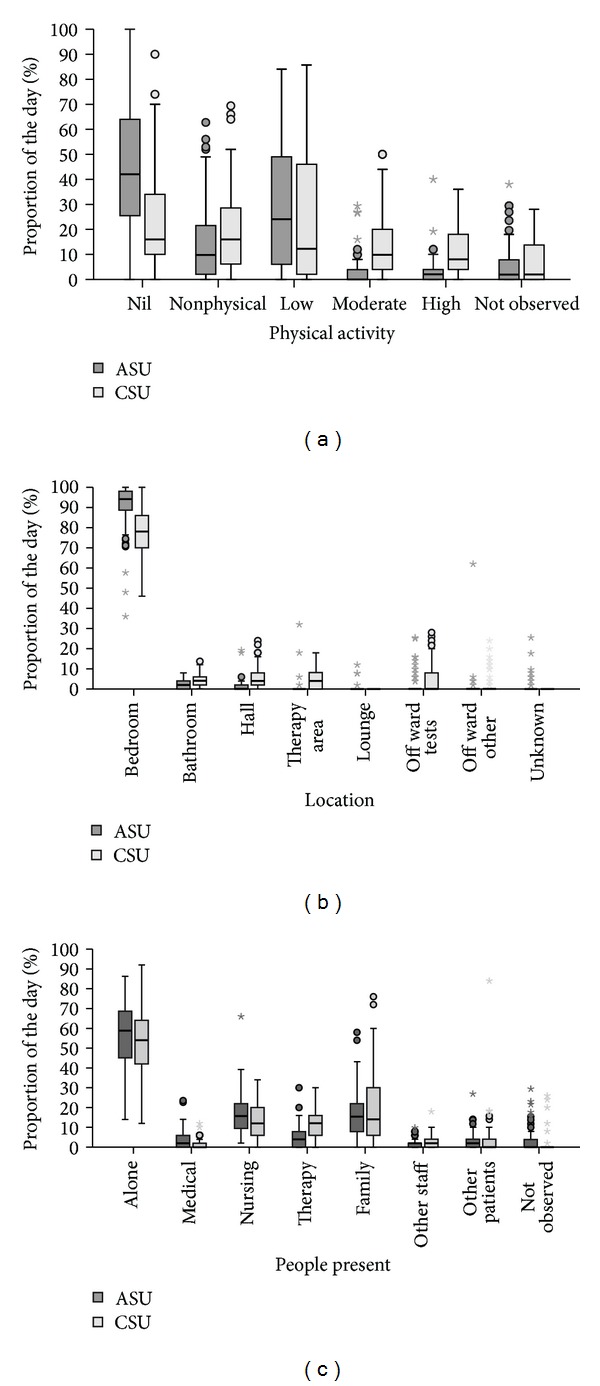
Patient activity in acute (ASU) and comprehensive (CSU) stroke unit care. Proportion of the day (a) in each physical activity category, (b) in each location, and (c) with different people present. Box: median and interquartile range (IQR). Whiskers: data within 1.5x IQR of lower and upper quartiles. Dots: data 1.5–3.0x IQR from lower and upper quartiles. Stars: data > 3.0x IQR from lower and upper quartiles. Therapy includes physiotherapy, occupational therapy, and speech therapy. People present categories are not mutually exclusive.

Patients in the CSU appeared to be more active than patients in the ASU (Figure 1(a)). The median proportion of the day spent in moderate or high level physical activities was 18.0% (IQR 8.0–35.0) for the CSU patients compared to only 3.8% (IQR 0.0–9.5) for the ASU patients. Conversely, ASU patients spent more time inactive or involved in nonphysical activities (ASU: median 58.8%, IQR 35.6–83.0; CSU: median 42.0%, IQR 20.0–63.0).

Using univariate median regression analyses, patients in the CSU spent an additional 14.1% of the day (95% CI: 9.3%–19.0%; *P* < 0.001) in moderate or high level activity, when compared with the ASU. Conversely, patients in the ASU spent an additional 16.8% of the day (95% CI: 4.7%–29.0%; *P* = 0.007) inactive or involved in nonphysical activity when compared with the CSU. After adjusting for differences in age, stroke severity, gender, days poststroke, and premorbid function, using multivariate median regression analyses, these findings remained significant. Furthermore, patients in the CSU spent 14.4% (95% CI: 8.9%–19.8%; *P* < 0.001) (adjusted) more of the day in moderate to high level activity and those in the ASU spent 18.5% (95% CI: 5.0%–32.0%; *P* = 0.008) (adjusted) more of the day inactive or involved in nonphysical activity.

#### 3.2.2. Location

Patients in the ASU appeared to spend more time in bedroom areas than patients in the CSU; however, in both units the majority of the day was spent in the bedroom (ASU: median 94.1%, IQR 88.6–98.1; CSU: median 78.0%, IQR 70.0–86.0) (Figure 1(b)). The median combined time spent in areas likely to promote activity, including the bathroom, hallway, therapy area, and off ward for purposes other than investigations, was only 3.8% (IQR 0.0–6.0) of the day for the ASU patients compared to 16.0% (IQR 10.0–24.0) of the day for CSU patients.

#### 3.2.3. People Present

In both units, patients spent more than half the day alone (ASU: median 58.8%, IQR 44.7–68.6; CSU: median 54.0%, IQR 41.0–64.0) (Figure 1(c)). The time spent with different people present was generally similar across sites; however, the CSU patients appeared to spend more time with a therapist present (physiotherapist, occupational therapist, or speech therapist) (ASU: median 3.8%, IQR 0.0–7.8; CSU: 12.0%, 6.0–16.2).

### 3.3. Formal Therapy Activity

The amount of physiotherapy and occupational therapy provided to patients in each unit is reported in Table 2. Consistent with the behavioural mapping data, patients in the CSU received significantly more physiotherapy time (*P* < 0.001) and more occupational therapy time per day (*P* < 0.001). The median total therapy time per day, combining both physiotherapy and occupational therapy, was 60.0 minutes (IQR 38.5–80.0) in the CSU compared to only 5.0 minutes (IQR 0.0–30.5) in the ASU. Thirty-six (49.3%) of the ASU patients did not receive any therapy from either physiotherapy or occupational therapy on the day of observation, compared to only 5 (6.8%) patients in the CSU.

### 3.4. First Mobilisation

Data for the time to admission and time to first mobilisation are summarised in Table 3. Complete data were not available for 21 (28.8%) of the ASU patients. Two of these patients had not yet been mobilised out of bed by the end of the day of observation, one of whom was four days poststroke and the other six days poststroke. The reason for bed rest was not provided. The time of stroke was not documented for three patients, the time of first mobilisation was not documented for 14 patients, and neither the time of stroke nor time of first mobilisation was documented for two patients.

Patients in the ASU had a significantly shorter time from stroke to admission. Despite the longer time to admission, patients in the CSU commenced mobilisation out of bed significantly earlier, from both time of stroke and time of admission, compared to patients in the ASU.

### 3.5. Patient Discharge

**Figure 2:**
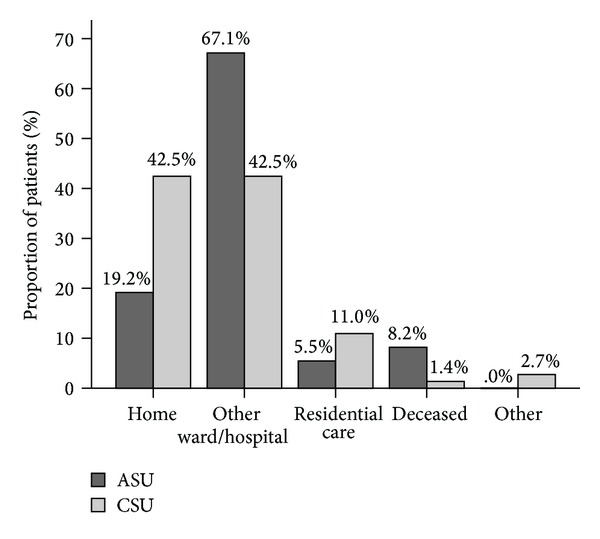
Discharge destination from acute (ASU) versus comprehensive (CSU) stroke unit care. Proportion of patients discharged to each destination.

The median length of stay was 13.0 days (IQR 8.0–19.5) for the ASU patients and 14.0 days (IQR 9.5–19.5) for the CSU patients. The discharge destinations for each hospital are illustrated in Figure 2. More patients were transferred to another ward or hospital in the ASU compared to the CSU. At both sites patients were usually transferred to another ward or hospital for the purpose of ongoing inpatient rehabilitation. More patients were discharged directly home from the CSU. Using univariate logistic regression analysis, the odds of discharge directly home was significantly higher from the CSU than the ASU (OR 3.1; 95% CI 1.5–6.5; *P* = 0.003). This result remained significant after adjusting for the effects of age, gender, stroke severity, and premorbid function (OR 3.7; 95% CI 1.4–9.5; *P* = 0.007).

## 4. Discussion

Early physical activity is considered a key feature of effective stroke care [2, 3] and preliminary evidence to support this intervention has emerged from two small randomised controlled trials of early mobilisation [4, 5]. In these studies early mobilisation was defined as mobilisation out of bed commencing within 24 hours of stroke and continuing frequent activity out of bed thereafter [4, 5]. However, the delivery of stroke care varies across different stroke services [22, 23] and as a consequence early physical activity levels are likely to vary between different models of acute stroke care. In the past, inconsistencies in behavioural mapping procedures and in the classification of physical activity have limited our ability to compare physical activity and care practices in hospitalised stroke patients across different units [24]. Studies which directly compare different stroke services provide a more robust evaluation of differences in physical activity in different models of stroke care; however, only two previous studies provide a direct comparison of early physical activity after stroke [13, 25]. These two studies are limited by small sample sizes, the timing of commencement of physical activity is not reported, and patient outcome was not evaluated. The current study directly compares early physical activity in two common models of stroke unit care.

The results of this study suggest that patients admitted to CSU care are more active within 14 days poststroke compared to patients admitted to ASU care. Patients in the CSU commenced activity out of bed sooner, received more therapy time, and spent more time away from bedroom areas, contributing to a greater level of physical activity. This finding is consistent with what would be expected in a rehabilitation model of care. These results support our recent review of ASU and CSU care, in which we found that the ASU model tends to focus primarily on acute medical care, while a stronger emphasis on multidisciplinary rehabilitation appears to exist in the CSU model even in the acute stage of stroke [10]. The greater emphasis on multidisciplinary rehabilitation in the CSU may promote the increased early physical activity found in the current study.

The results of the current study are also consistent with a previous study, in which patients admitted to a CSU in Trondheim, Norway, were found to be more active within 14 days poststroke than patients in five Melbourne stroke units, four of which were ASUs [13]. The Trondheim and Perth CSUs share similar characteristics which may contribute to the increased early activity levels in these units, including an increased focus on early intensive rehabilitation, policies and procedures that promote early mobilisation and avoidance of bed rest, staff training and education in early mobilisation, and the provision of equipment and a physical environment which encourage activity [13, 21].

In contrast to the rehabilitation focus in the Perth and Trondheim CSUs, in a previous study of physical activity in five stroke units the authors reported that in the ASUs which they observed the staff considered that their main role was to assess new patients and that patients suitable for discharge directly home should be the main priority for rehabilitation interventions [26]. This approach likely contributed to the lower levels of therapy and activity in the Melbourne ASU in this study. The issue of the physical environment in which care is delivered may also influence activity. In the Melbourne ASU observed in the current study, en-suite bathrooms were present in most bedroom areas which may have limited opportunities for walking to and from the bathroom. In contrast, the Perth unit had bathroom areas that were located separately from the bedroom areas, providing opportunities for functional, goal directed mobility. Access to wheelchairs for patients unable to ambulate was limited in the Melbourne ASU, reducing the amount of time patients were able to sit out of bed and making transport outside of bedroom areas more difficult. Despite the location of physiotherapy and occupational therapy areas and a large lounge area nearby to the ward, these areas were not frequently used by patients. Further formal evaluation of the environment, both in terms of ward layout, single bed, or multibed rooms and distances between key areas of activity, would be worthwhile in future studies to better assess the potential contribution of these to activity.

Staffing levels in the Melbourne ASU were similar to those in the Perth CSU. Both units were staffed with a nurse-patient ratio of 1 : 4 and an occupational therapist-patient ratio of approximately 1 : 13, as per the Perth CSU. Physiotherapy staffing levels in the Melbourne ASU were lower than the Perth CSU, with a physiotherapist-patient ratio of approximately 1 : 16 in Melbourne compared to 1 : 11 in the Perth CSU; however, this alone would not account for the fact that the median combined therapy time in the CSU was 12 times that of the ASU.

In addition to the increased early physical activity levels, the results of this study suggest that patients are also more likely to be discharged directly home from the CSU compared to the ASU. Although the median length of stay was one day shorter in the ASU than in the CSU, any economic benefit from this shorter length of stay is likely to have been lost due to the costs of the increased need for inpatient rehabilitation beyond the acute period. While the results of this study do not establish a causal relationship between early physical activity and discharge destination, it does raise the question as to whether a greater focus on early intensive rehabilitation, an earlier commencement of activity out of bed, and an increased level of physical activity early after stroke, may improve the likelihood of discharge home. In a previous randomised controlled trial comparing CSU care to stroke care on a general medical ward, the results of a multivariate analysis revealed that an earlier start to mobilisation out of bed was the most important factor associated with an increased likelihood of discharge home within six weeks [2]. Furthermore, in a randomised controlled trial comparing early mobilisation to standard care after stroke, the utilisation of rehabilitative services including inpatient rehabilitation was considerably less in the subacute stage for the early mobilisation group, contributing to significant cost savings [27]. However, it is possible that patients who are expected to be discharged directly home may have been considered a higher priority for rehabilitation interventions including the promotion of early physical activity. Therefore, activity levels in the CSU may have been higher because more patients were discharged directly home. This is supported by the findings of a recent study investigating clinical prioritisation by acute stroke clinicians indicating that planned discharge destination may actually be a driver of quality of care [28], suggesting that discharge destination may have an impact on early physical activity levels, rather than the other way around. It is also possible that the difference in discharge destinations between the two units in the current study may have been the result of differences in processes of care other than early mobilisation. In addition, factors such as differences in the availability of ongoing inpatient and outpatient rehabilitation, early supported discharge programs, and community-based formal care services, may have also influenced discharge destination, particularly given that the two units observed were in different states of Australia and therefore under different systems of healthcare.

The observational design of this study gives rise to a number of limitations, including the potential for observer bias and the possibility that staff and patient behaviour were influenced by the presence of the observer. However, a standardised observation technique was used to reduce observer bias and observers attempted to remain inconspicuous at all times so as to minimise any influence on staff and patient behaviour. The intermittent nature of the behavioural mapping method may have overestimated or underestimated patient activity; however, continuous observation would not have been feasible with the behavioural mapping and this method has the advantage of allowing patient location and the people present to be observed, in addition to patient activity.

The accuracy of the first mobilisation data may also be limited given that this data was determined from the medical record. For a number of patients the exact time of stroke or first mobilisation out of bed was not documented in the medical record and it is possible that this information may have been incorrectly documented for other patients. However, this was the most accurate means available for acquiring this information given that patients could be recruited to the study some days after they were first mobilised.

The lack of randomisation and the heterogeneity of the patient groups is a further limitation of this study. While it would be ideal to compare different stroke unit models in a randomised controlled trial, there are logistical issues with randomising patients to stroke units at different sites; therefore, other research methods such as the observational design of this study are required. To account for the lack of randomisation and the heterogeneity of the patient groups we matched patients on the basis of age, stroke severity (NIHSS), premorbid function (premorbid mRS > 2), and ambulation status on the day of observation (MSAS Gait < 6). Despite this matching process significant differences remained between the two groups for gender, OCSP infarct classification, and premorbid mobility. However, as part of our analysis we adjusted for gender and while we did not specifically adjust for OCSP infarct classification or premorbid mobility, we did adjust for stroke severity which we considered to be a proxy variable for OCSP classification and we also adjusted for premorbid function which we considered a proxy variable for premorbid mobility.

While the matching process does increase internal validity, it does have the potential to reduce external validity and therefore the generalisability of our findings. Furthermore, the generalisability of the results is also limited by the investigation of only two centres from one country in this study. Larger multicenter studies are required to enable better generalisation.

## 5. Conclusions

Evidence to support the implementation of any one model of stroke unit care over another is lacking. We have found that a stroke unit model which incorporates both acute care and rehabilitation is associated with increased early physical activity and an increased likelihood of discharge directly home, in comparison to the model which provides acute care only. However, early physical activity is just one component of stroke unit care and further research is required which compares key features of different models and which provides a more extensive comparison of short-term and long-term outcomes.

## Figures and Tables

**Table 1:** Patient characteristics.

	ASU	CSU	*P* value
*N*	73	73	
Age			
Median (IQR)	78.8 (66.1–83.7)	75.5 (65.9–81.5)	0.21
Gender—*n* (%)			
Male	35 (47.9)	51 (69.9)	
Female	38 (52.1)	22 (30.1)	0.01
First stroke—*n* (%)			
Yes	54 (74.0)	58 (79.5)	
No	18 (24.7)	15 (20.5)	
Missing	1 (1.4)	0 (0.0)	0.56
Days poststroke			
Median (IQR)	6.0 (4.0–8.5)	7.0 (4.5–9.0)	0.31
Stroke type—*n* (%)			
Infarct	61 (83.6)	59 (80.8)	
Haemorrhage	11 (15.1)	14 (19.2)	
Missing	1 (1.4)	0 (0.0)	0.66
NIHSS			
Median (IQR)	6.0 (4.0–12.0)	6.0 (4.0–10.0)	0.58
OCSP infarct classification—*n* (%)			
TACI	13 (17.8)	11 (15.1)	
PACI	29 (39.7)	19 (26.0)	
POCI	9 (12.3)	6 (8.2)	
LACI	6 (8.2)	23 (31.5)	
Missing	5 (6.8)	0 (0.0)	0.01
Side of lesion—*n* (%)			
Left	30 (41.1)	31 (42.5)	
Right	41 (56.2)	39 (53.4)	
Brainstem	1 (1.4)	3 (4.1)	
None evident/unknown	1 (1.4)	0 (0.0)	0.70
Premorbid MRS—*n* (%)			
Independent (0–2)	56 (76.7)	58 (79.5)	
Dependent (>2)	17 (23.3)	15 (20.5)	0.84
Prestroke accommodation—*n* (%)			
Home alone	21 (28.8)	26 (35.6)	
Home with someone	48 (65.8)	43 (58.9)	
Residential care	3 (4.1)	3 (4.1)	
Other	1 (1.4)	1 (1.4)	0.82
Prestroke mobility—*n* (%)			
Independent no aids	51 (69.9)	64 (87.7)	
Independent with aid	19 (26.0)	9 (12.3)	
Walking with supervision	3 (4.1)	0 (0.0)	0.01
MSAS Gait—*n* (%)			
Independent	16 (21.9)	18 (24.7)	
Not independent	57 (78.1)	55 (75.3)	0.85

NIHSS: National Institutes of Health Stroke Scale; OCSP: Oxfordshire Community Stroke Project; TACI: total anterior circulation infarct; PACI: partial anterior circulation infarct; POCI: posterior circulation infarct; LACI: lacunar infarct; MRS: modified Rankin Score; MSAS: mobility scale for acute stroke patients.

**Table 2:** Amount of therapy provided in acute (ASU) versus comprehensive (CSU) stroke unit care.

	ASU	CSU
	*N* = 73	*N* = 73
*Physiotherapy *		
Patients treated—*n* (%)	32 (43.8)	62 (84.9)
Therapy minutes per day		
Median (IQR)	0.0 (0.0–19.5)	36.0 (22.0–50.0)
Range	0–116	0–105
Therapy minutes per session		
Median (IQR)	20.0 (11.5–33.7)	40.0 (26.0–50.0)
Range	5–65	5–90
Frequency of therapy sessions per day—*n* (%)		
None	41 (56.2)	11 (15.1)
One	30 (41.1)	55 (75.3)
Two	2 (2.7)	7 (9.6)

*Occupational therapy *		
Patients treated—*n* (%)	16 (21.9)	48 (65.8)
Therapy minutes per day		
Median (IQR)	0.0 (0.0-0.0)	20.0 (0.0–40.0)
Range	0–60	0–100
Therapy minutes per session		
Median (IQR)	29.5 (20.0–35.0)	30.0 (20.0–40.0)
Range	10–60	5–80
Frequency of therapy sessions per day—*n* (%)		
None	57 (78.1)	25 (34.2)
One	16 (21.9)	44 (60.3)
Two	0 (0.0)	4 (5.5)

**Table 3:** Time to first mobilisation in acute (ASU) versus comprehensive (CSU) stroke unit care.

	ASU	CSU	*P* value
Stroke to admission (hours)			
*N*	68	73	
Median (IQR)	3.6 (1.5–7.6)	6.4 (2.1–18.1)	0.004
Range	0.0–83.7	0.8–106.0	
Stroke to mobilisation (hours)			
*N*	52	73	
Median (IQR)	51.0 (27.0–76.7)	32.0 (24.2–52.8)	0.015
Range	2.2–249.5	5.2–209.0	
Admission to mobilisation (hours)			
*N*	55	73	
Median (IQR)	28.4 (21.3–67.6)	20.6 (12.6–38.3)	0.000
Range	0.3–248.2	1.9–206.6	

*Mann-Whitney *U* test.
